# Unveiling the Chemical Composition, Enantiomeric Profile, Antibacterial, Anticholinesterase and Antioxidant Activity of the Essential Oil of *Aloysia triphylla* Royle

**DOI:** 10.3390/molecules30132849

**Published:** 2025-07-03

**Authors:** Cinthia Mejia-Ramos, Julio Reynaldo Ruiz-Quiroz, Maria Elena Salazar-Salvatierra, James Calva, Eddie Loyola-Gonzales, Haydee Chávez, Javier Hernán Chavez-Espinoza, Josefa Bertha Pari-Olarte, José Santiago Almeida-Galindo, Oscar Herrera-Calderon

**Affiliations:** 1 Faculty of Pharmacy and Biochemistry, Universidad Nacional Mayor de San Marcos, Lima 15001, Peru; cinthiamejiaramos@gmail.com; 2 Institute for Research in Biological Chemistry, Microbiology and Biotechnology “Marco Antonio Garrido Malo”, Faculty of Pharmacy and Biochemistry, Universidad Nacional Mayor de San Marcos, Lima 15001, Peru; jruizq@unmsm.edu.pe (J.R.R.-Q.); msalazars@unmsm.edu.pe (M.E.S.-S.); 3 Departamento de Química, Universidad Técnica Particular de Loja (UTPL), Loja 1101608, Ecuador; jwcalva@utpl.edu.ec; 4 Department of Pharmaceutical Science, Faculty of Pharmacy and Biochemistry, Universidad Nacional San Luis Gonzaga, Ica 11001, Peru; eddie.loyola@unica.edu.pe; 5 Department of Pharmaceutical Chemistry, Faculty of Pharmacy and Biochemistry, Universidad Nacional San Luis Gonzaga, Ica 11001, Peru; hchavez@unica.edu.pe (H.C.); javierchavez@unica.edu.pe (J.H.C.-E.); bertha.pari@unica.edu.pe (J.B.P.-O.); 6 Department of Basic Sciences, Faculty of Human Medicine, Universidad Nacional San Luis Gonzaga, Ica 11001, Peru; santiago.almeida@unica.edu.pe; 7 Department of Pharmacology, Bromatology and Toxicology, Faculty of Pharmacy and Biochemistry, Universidad Nacional Mayor de San Marcos, Lima 15001, Peru

**Keywords:** volatile oil, bioactivity, lemon verbena, antimicrobial, Ach, GC-MS

## Abstract

*Aloysia triphylla* is widely used in traditional medicine from Peru for its sedative, digestive and anti-inflammatory properties. However, comprehensive studies on the biological activities of its essential oil (EO), particularly from Peruvian sources, remain limited. This study aimed to analyze the chemical composition and enantiomeric profile of *A. triphylla* EO and evaluate its antibacterial, antioxidant, anticholinesterase, and cytotoxic activities. The EO was obtained by steam distillation and analyzed using gas chromatography–mass spectrometry (GC-MS). A total of 62 compounds were identified, with (*E*)-caryophyllene (16.80%), *β*-pinene (9.96%), and germacrene D (10.00%) being the major components. Enantiomeric analysis revealed specific chiral signatures, including (−)-*α*-pinene, (+)-limonene, and (*R*)-(−)-linalool. The EO exhibited significant antibacterial activity, particularly against *Bacillus subtilis* (MIC = 5 µg/mL), and weak antioxidant activity (IC_50_ = 7720 and 4648 µg/mL for DPPH and ABTS, respectively). Additionally, the EO demonstrated moderate acetylcholinesterase inhibition (IC_50_ = 87.8 µg/mL) and cytotoxicity in the *Artemia salina* assay (LC_50_ = 964 µg/mL). These findings suggest that *A. triphylla* EO possesses promising bioactivities with potential applications in pharmaceutical and cosmetic fields.

## 1. Introduction

Infectious diseases are the leading cause of death worldwide, and antibiotics prescribed to combat them are acquiring high resistance [1]. However, while antimicrobial resistance occurs naturally, the public health crisis also leads to problems, such as the overuse of antibiotics, their accumulation in the environment, and their application in the animal and food industries [2]. Additionally, the high prevalence of antimicrobial resistance in pathogenic microorganisms is a major challenge to global public health, compromising treatment efficacy, and contributing to the persistence and recurrence of infections [3]. Therefore, there has been growing interest in identifying new antimicrobial agents derived from natural products, including plants, bacteria, fungi, and marine and animal sources. On the other hand, essential oils (EOs) have garnered attention due to their antibacterial activity and ability to target multiple cellular structures and functions in microorganisms [4]. The discovery of novel antibacterial compounds is critical for the development of new therapeutic strategies and preservative agents applicable in the pharmaceutical, cosmetic, and food industries.

Oxidative stress plays a key role in the pathogenesis of several chronic diseases. The accumulation of reactive oxygen species (ROS) in living organisms, including hydroxyl radicals, hydrogen peroxide, superoxide anions, and singlet oxygen, generated during normal cellular respiration particularly during the incomplete reduction of oxygen within the mitochondria, can result in significant cellular damage [5]. Natural antioxidants, especially EOs, have been increasingly investigated for their capacity to mitigate oxidative stress, and some EOs have demonstrated interesting activities, including those rich in oxygenated monoterpenes and sesquiterpenes [6].

Recently, there has been growing interest in EOs for their potential to inhibit acetylcholinesterase (AChE), an enzyme responsible for modulating the neurotransmitter acetylcholine. AChE inhibitors are vital in treating symptoms of neurodegenerative conditions such as Alzheimer’s disease, which are characterized by a significant decline in cholinergic function [7]. Synthetic AChE inhibitors, such as donepezil, rivastigmine, and galantamine, are commonly used to enhance cognitive function and slow disease progression. However, these medications often have side effects, including gastrointestinal problems, liver toxicity, and diminishing effectiveness over time [8]. Consequently, there is increasing interest in identifying natural AChE inhibitors from plants, including essential oils [9], which may provide safer alternatives with a range of biological activities. Therefore, assessing the anticholinesterase potential of *A. triphylla* EO is part of an ongoing effort to discover new neuroprotective agents from natural sources.

*Aloysia triphylla* Royle (Synonym: *Aloysia citrodora*), also known as “cedrón” (in Spanish), is an aromatic plant in the *Verbenaceae* family. It is native to South America (Peru, Argentina, and Bolivia), is widely distributed in tropical and subtropical regions, and is traditionally used to treat gastrointestinal, neurological, and respiratory diseases through aerial part (leaves and flowers) infusions [10]. In Peru, *A. triphylla* grows in the Andes at an altitude of approximately 2000 to 3000 m, although it also grows in the coastal region. The chemical composition of EO is abundant in monoterpenes and sesquiterpenes, and is highly influenced by environmental factors such as altitude, climate, and soil conditions, which might affect its biological activities [11]. For instance, in a study in Palestine, geranial, neral, and curcumene were identified as the main volatile components [12]. The EO from other regions was abundant in limonene and citral [13,14]. These differences may affect the antibacterial and antioxidant activities.

Based on previous studies, this study aimed to determine the antibacterial, antioxidant, and anticholinesterase activities of *A. triphylla* EO obtained from Peru by steam distillation. The chemical composition of the volatile components was assessed using gas chromatography–mass spectrometry (GC-MS). In vitro antioxidant activity was assessed through DPPH (2,2-diphenyl-1-picrylhydrazyl) and 2, 2′-azino-bis (3-ethylbenzothiazoline-6-sulfonic acid) (ABTS) assays. Antibacterial activity was evaluated against both Gram-positive and Gram-negative strains using a resazurin-based colorimetric microdilution assay. Anticholinesterase activity was assessed spectrophotometrically using the Ellman method, and the lethal concentration 50 (LC_50_) was determined using the toxicity model of *Artemia salina*.

## 2. Results

### 2.1. Chemical Characterization of Essential Oil

**Figure 1:**
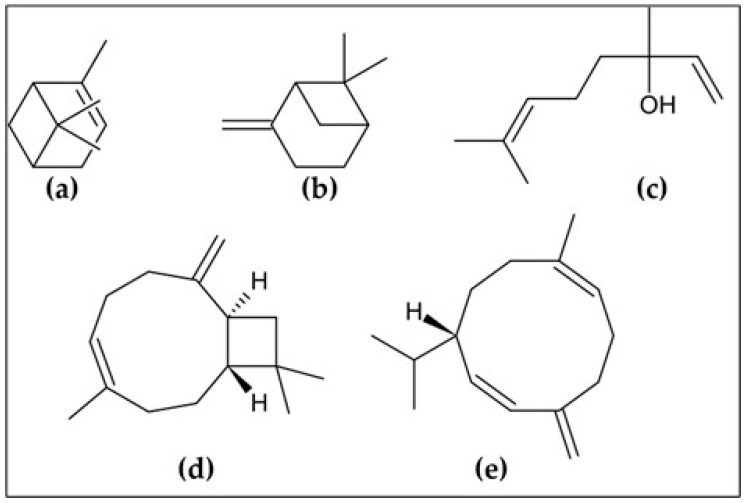
The most abundant compounds of *A. triphylla* EO: (**a**) *α*-pinene; (**b**) *β*-pinene; (**c**) linalool; (**d**) (*E*)-caryophyllene; and (**e**) germacrene D.

**Figure 2:**
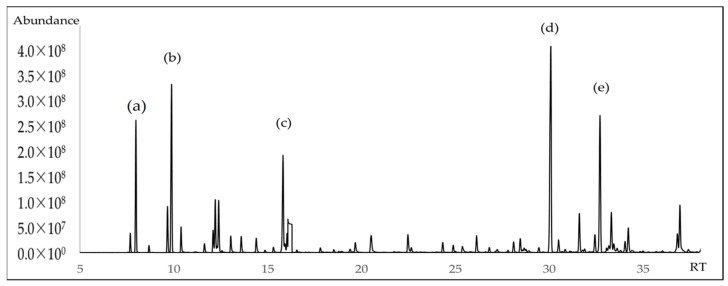
GC-MS chromatogram of *A. triphylla* EO on a nonpolar DB5-ms column (**a**) *α*-pinene; (**b**) *β*-pinene; (**c**) linalool; (**d**) (*E*)-caryophyllene; (**e**) Germacrene D.

The chemical composition of *A. triphylla* EO revealed the presence of 62 compounds, representing 99.84% of the total EO content (Table 1). Figure 1 and Figure 2 show the abundant compounds identified as (*E*)-caryophyllene (16.80 ± 1.00%), *β*-pinene (9.96 ± 0.95%), *α*-pinene (6.71 ± 0.85%), linalool (6.30 ± 0.90%), and germacrene D (10.00 ± 0.95%).

### 2.2. Enantiomeric Distribution of A. triphylla Essential Oil

The enantiomeric analysis performed in this study by gas chromatography coupled to mass spectrometry (GC-MS) allowed the identification of the stereoisomeric profile of the essential oil of *A. triphylla* (Table 2 and Figures S1–S7). This analysis revealed the presence of three pairs of enantiomers, namely (1*S*,5*S*)-(−)-*α*-pinene and its enantiomer (1*R*,5*R*)-(+)-*α*-pinene, (1*S*,5*S*)-(−)-*β*-pinene and its enantiomer (1*R*,5*R*)-(+)-*β*-pinene, and (*R*)-(−)-linalool enantiomer with its enantiomer (*S*)-(+)-linalool, and three chiral compounds present exclusively in one of their isomers, namely (1*S*,5*S*)-(−)-sabinene, (*R*)-(+)-limonene, and (*R*)-(+)-germacrene D. 

### 2.3. Antibacterial Activity of the A. triphylla EO

The antibacterial potential of *A. triphylla* essential oil was evaluated against four bacterial strains using the colorimetric microdilution method to determine the minimum inhibitory concentration (MIC). The results revealed notable variations in susceptibility among microorganisms, as shown in Table 3. The EO exhibited a moderate inhibitory effect against *S. aureus* and *B. subtilis*, with MIC values of 80 ± 2.00 µg/mL and 5 ± 1.00 µg/mL, respectively. However, a significantly higher MIC was observed for *E. coli* (320 ± 0.58 µg/mL), suggesting the reduced susceptibility of this Gram-negative bacterium. *P. aeruginosa* displayed the highest resistance, as no inhibition was observed at the tested concentrations (MIC > 500 µg/mL). Ciprofloxacin, which was used as a positive control, demonstrated considerably lower MIC values across all bacterial strains, confirming its superior antibacterial potency. Statistical analysis through two-way ANOVA indicated significant differences (*p* < 0.0001) in antibacterial activity between treatments. Additionally, Sidak’s multiple comparison test confirmed the statistical significance of these differences across bacterial strains.

### 2.4. Antioxidant Activity of A. triphylla EO

Table 4 shows the antioxidant capacity of *A. triphylla* EO, evaluated using DPPH and ABTS radical scavenging assays. The results showed markedly lower antioxidant activity than that of the Trolox standard, as indicated by the significantly higher IC_50_ values for the essential oil in both assays. Nonetheless, the calculated TEAC values demonstrate that the oil possesses a measurable antioxidant potential. Statistical analysis confirmed that the differences between the EO and Trolox controls were highly significant (*p* < 0.0001) for both methods.

### 2.5. Acetylcholinesterase Inhibition of A. triphylla EO

**Figure 3:**
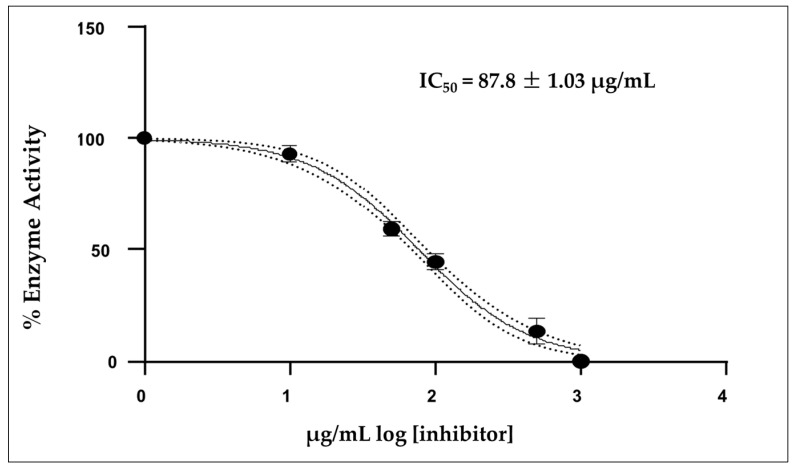
Acetylcholinesterase activity of IC_50_ concentration of the essential oil *A. triphylla* EO from Perú; Mean ± SD (*n* = 3).

Figure 3 shows that the mean inhibitory concentration (IC_50_) of the EO was 87.8 ± 1.03 µg/mL, which was calculated from three independent measurements. Donepezil was used as a positive control, showing a IC_50_ value of 12.40 µg/mL under the same experimental conditions.

### 2.6. Cytotoxicity of A. triphylla EO

The brine shrimp lethality assay was used to evaluate the cytotoxic potential of *A. triphylla* EO. The test revealed moderate toxicity, as indicated by the LC_50_ value shown in Table 5. In contrast, the solvent control showed no toxic effects, confirming the reliability of the assay. Potassium dichromate, which was used as a positive control, showed high toxicity, as expected, validating the sensitivity of the method.

## 3. Discussion

Phytochemical analysis revealed that the EO contained three major components, which were identified as (*E*)-caryophyllene (16.80 ± 1.00%), germacrene D (10.00 ± 0.95%) and *β*-pinene (9.96 ± 0.95%). However, our findings differ from those obtained in studies on the content of volatile components; the essential oil of *A. citrodora* from Portugal contained citral isomers geranial (18.7–21.1%) and neral (15.3–16.2%) as the main compounds [14]. In a study on *A. citrodora* from Algeria, the major compounds were citral (13.80%), D-limonene, (12.16%), and neral (10.67%) [15]. EO obtained by the hydrodistillation of leaves and inflorescences from Brazil contained two compounds, *β*-pinene (22.1%) and trans-pinocamphone (13.1%) [16]. Another sample from Brazil showed that the concentration of volatile components can vary according to the season, with limonene and citral being the main constituents. However, in some seasons, caryophyllene and/or caryophyllene oxide were detected in significant concentrations [17]. This variation might be explained by several factors (weather, growing conditions, cultivation conditions, and harvest season) that influence the contents of caryophyllene and *β*-pinene instead of limonene or citral, which are commonly found in *A. triphylla* species from other regions. A study conducted in Brazil revealed that winter was the best season for detecting *α*-citral, limonene, and *β*-citral [18]. In contrast, in some species of aromatic plants such as *Rhodomyrtus tomentosa*, *α*-pinene and *β*-caryophyllene were found in higher concentrations in young leaves than in older leaves [19]. Similar to our report, our samples were abundant in months with rainy weather and temperatures ranging between 15 and 20 °C, and only young leaves were used in this study. For the first time, this study identified the stereoisomeric profile of *A. triphylla* essential oil using GC-MS with a chiral column, which is relevant for assessing the authenticity and purity of the oil, because the enantiomers of chiral compounds can vary according to botanical origin, environmental conditions, or adulteration [20].

The antioxidant activity of our EO was similar to that obtained using the DPPH and ABTS methods. In other investigations, EOs obtained using the Clevenger apparatus and microwave-assisted hydrodistillation varied in their antioxidant activity against DPPH, with values of 9.583 ± 0.005 mg/mL and 8.631 ± 0.005 mg/mL, respectively [14]. However, these results are similar to our findings. Hashemi et al. investigated the extraction of EO from *A. citrodora* and reported EC_50_ values ranging from 5 to 10 mg/mL and 10 to 15 mg/mL for hydrodistilled and ultrasound-assisted extraction, respectively [21]. In another study, EO from Jordan did not show antioxidant activity against DPPH [22]. The limited antioxidant activity of our EO might be explained by the presence of low-oxygenated sesquiterpenes in contrast to oxygenated monoterpenes, which tend to have limited radical scavenging capacity [23]. Furthermore, the presence of a phenolic group containing an electron-repelling group at the ortho position to the phenolic group is required to achieve a strong radical scavenging effect [24,25].

In contrast, antibacterial activity was found to be effective against Gram-positive bacteria, especially *B. subtilis*. Previous studies have reported the antibacterial activity of EO against important clinical strains, such as *S. aureus* and *E. coli* [26]. In other studies, *A. triphylla* EO from Brazil was shown to be effective against six bacterial strains. It was particularly effective against *Enterococcus faecium* ATCC 10541 (MIC = 0.05 mg/mL), *B. subtilis* (MIC = 0.50 mg/mL) and *Salmonella cholerasuis* (MIC = 0.60 mg/mL), but presented moderate inhibition against *S. aureus* (MIC = 0.80 mg/mL), with the major components detected being neral and geranial [27]. In another study, *A. triphylla* EO from Argentina exhibited antibacterial activity against a wide range of microorganisms, including *S. aureus*, *Staphylococcus epidermidis*, *B. cereus*, *Micrococcus luteus*, *E. faecalis*, *E. coli*, *Klebsiella sp*., and *Proteus mirabilis*. Notably, it was one of the most effective compounds against *B. cereus* and *S. aureus*, with MICs of 56.25 µg/disk for both bacteria. However, it was not effective against *P. aeruginosa*, which appeared to be the most resistant microorganism in this study [28]. According to our results and those of other investigations, *A. triphylla* EO exhibits promising antibacterial properties against a wide range of bacteria, particularly against gram-positive strains. These findings suggest that this EO possesses potential for application used in food preservation and as an alternative treatment for infections caused by Gram-positive bacteria.

The lipophilic nature of EOs allows them to penetrate and disrupt the cell membrane structure, thereby affecting their integrity and function [29]. Additionally, the cell wall structure of Gram-positive bacteria facilitates the penetration of hydrophobic molecules, allowing them to act on both the cell wall and the cytoplasm. Phenolic compounds, also found in EOs, typically exhibit antimicrobial activity against Gram-positive bacteria and are likely to be more susceptible to terpene activity [30]. Interestingly, EOs often demonstrate stronger antibacterial activity against Gram-positive bacteria than Gram-negative bacteria. This is likely due to the simpler cell wall structure of Gram-positive bacteria, which lacks the outer membrane present in Gram-negative bacteria, making them more susceptible to EO penetration [31]. Regarding the most abundant components in our EO, compared to (+)-*α*-pinene, (+)-*β*-pinene was two to twelve times more efficient against both Gram-positive and Gram-negative bacteria, according to Van Zyl et al. [32], and (*E*)-caryophyllene showed more antimicrobial activity towards Gram-positive bacteria than Gram-negative bacteria, showing no inhibition of *P. aeruginosa* [33].

The essential oil of *A. triphylla* showed moderate inhibitory activity on AChE, with an IC_50_ of 87.8 µg/mL, indicating that a relatively high amount of oil is needed for 50% enzyme inhibition compared to the drug donepezil with an IC_50_ of 12.40 µg/mL, designed specifically for this purpose. This finding is relevant in the context of the search for natural compounds with potential applications in the treatment of neurodegenerative diseases, such as Alzheimer’s disease, where the reversible inhibition of AChE may help maintain adequate levels of acetylcholine in the central nervous system [34]. Several studies have shown that some of these compounds can directly interact with AChE. (*E*)-*β*-Caryophyllene has previously been reported to inhibit AChE from *Electrophorus electricus* at a concentration of 0.06 mM of 32% [34]. Linalool, for example, has shown the ability to inhibit AChE in vitro [35]. *β*-pinene and *α*-pinene have also been linked to neurological activity [36]. It should be noted that the activity observed in this study is probably the result of a synergistic effect between multiple compounds present in the oil, rather than the individual action of just one of them [37].

*Artemia salina* has been widely used as a model organism for assessing the eco-toxicity of various substances, including EOs. The brine shrimp *Artemia salina* offers numerous advantages as a toxicity model, including simplicity, low cost, and reproducibility [38]. EOs from Argentina, such as *Aloysia polystachia* and *A. triphylla*, had values ranging between LC_50_ = 6459 µg/mL and LC_50_ = 1279 µg/mL, respectively [39]. In our study, the LC_50_ of the essential oil showed a value of 964.0 ± 20.6 µg/mL, which is below 1000 µg/mL and is considered a moderate cytotoxic [40]. However, it is important to note that these results should be considered preliminary, and may require validation using other toxicity models or in vivo studies.

## 4. Materials and Methods

### 4.1. Chemicals

Resazurin sodium salt, potassium dichromate, potassium persulfate, 2,2-diphenyl-1-picrylhydrazyl (DPPH), 2,2′-azino-bis (3-ethylbenzothiazoline-6-sulfonic acid (ABTS), dimethylsulfoxide (DMSO), and Trolox (6-hydroxy-2,5,7,8-tetramethylchroman-2-carboxylic acid) were purchased from Sigma-Aldrich (St. Louis, MO, USA). Methanol and other solvents were purchased from Merck-Peruana (Lima, Peru).

### 4.2. Plant Material and Essential Oil Obtention

Young leaves of *A. triphylla* were collected from the district of Aucará, province of Lucanas, Department of Ayacucho, Peru. The collection area was located at an altitude of approximately 3231 m.a.s.l. The geographical coordinates of the collection site were 14°16′52″ S, 73°58′29″ W, and sample collection was conducted between January and March 2022 during the rainy season. The material plant was identified by the botanist Hamilton Beltran Santiago at the herbarium of the Universidad Nacional Mayor de San Marcos (Id. 051-2022-USM-MHN). Extraction was performed in a Clevenger-type apparatus for 3 h. The EO yield was 0.95%.

### 4.3. Determination of Essential Oil Composition

The volatile components of the essential oil *A. triphylla* were identified using a gas chromatography–mass spectrometry (GC) model Trace 1310 supplied by Thermo Fisher Scientific (Waltham, MA, USA) coupled with a single quadrupole mass spectrometer (MS) model ISQ 7000; all EOs were analyzed using a non-polar capillary column based on 5% phenyl-methylpolysiloxane (30 m × 0.25 mm, 0.20 μm film thickness, Agilent Technologies, Santa Clara, CA, USA). The samples were injected in split mode (40:1) by introducing 1 µL into 1000 µL of cyclohexane solution (1:100). The volatile components of the EOs were identified by comparing the relative retention indices (LRI) and NIST 23 Mass Spectra Library [41]. The LRIs were calculated and compared with a homologous series of n-alkanes C9-C25 (Sigma–Aldrich, St. Louis, MO, USA) [42], as shown in Figure S8.

### 4.4. Enantioselective Profile of the Essential Oil

Enantioselective analysis was performed using gas chromatography–mass spectrometry (GC-MS) with an enantioselective column based on 2,3-diethyl-6-tert-butyldimethylsilyl-β-cyclodextrin (25 m × 0.25 mm, 0.25 µm film thickness, from Mega, Milan, Italy). The injector temperature and carrier gas flow rate were the same conditions as those used in the GC-MS analysis. The injector was operated in the split mode (ratio of 50:1) to enhance the separation and detection of enantiomeric compounds. The oven temperature program started at 60 °C and was held for 2 min, followed by a gradual increase of 2 °C/min to 220 °C. Linear retention indices (LRIs) were calculated by injecting a standard mixture of n-alkanes C9–C25 (Sigma–Aldrich, St. Louis, MO, USA), following the methods of Van den Dool and Kratz. The enantiomers were identified based on their characteristic mass spectra and elution order, which were confirmed using commercially available, enantiomerically pure reference standards [43].

### 4.5. Evaluation of the Antibacterial Activity

The antibacterial activity of *A. triphylla* EO was evaluated using the microdilution method and the colorimetric detection of resazurin to determine the Minimum Inhibitory Concentration (MIC). The test was conducted against four reference bacterial strains, *Escherichia coli* ATCC 25922, *Staphylococcus aureus* ATCC 25923, *Bacillus subtilis* ATCC 6633, and *Pseudomonas aeruginosa* ATCC 27853. A stock solution of EO was prepared in Mueller-Hinton broth, with ten two-fold serial dilutions generated. The ciprofloxacin standard served as the positive control, following CLSI guidelines, and was prepared at an initial concentration of 640 µg/mL. Resazurin was used as a redox indicator for colorimetric detection. A volume of 0.03 mL of the resazurin solution was added to 6 mL of the 2× inoculum suspension, ensuring a visible color change in the presence of bacterial growth. Bacterial inoculum was prepared by culturing the strains on Tryptic Soy Agar at 37 °C for 24 h. Colonies were suspended in 0.9% saline, adjusted to a 0.5 McFarland standard (1–2 × 10^8^ CFU/mL), and further diluted with Mueller–Hinton broth to obtain a final concentration of 1–5 × 10^5^ CFU/mL. The assay was carried out in sterile 96-well microplates, in which 100 µL of EO dilution and 100 µL of resazurin-inoculated bacterial suspension were added to each well. The plates were then incubated at 37 °C for 24 h. MIC values were determined as the lowest concentration that prevented a color change from blue to pink or colorless. All assays were performed in triplicate.

### 4.6. Antioxidant Activity

In the DPPH assay, EO was diluted in methanol and tested at various concentrations (0–2000 µg/mL). The DPPH solution was prepared using methanol at a final concentration of 0.001 mM. Next, 100 µL of EO was reacted with 900 µL of DPPH for 30 min in the dark at room temperature. Finally, absorbance was measured at 517 nm using a UV-VIS Genesys 150 (Thermo Scientific, Waltham, MA, USA) spectrophotometer. Trolox was used as the reference antioxidant standard. The percentage of DPPH radical inhibition was calculated, and the IC_50_ (the concentration that inhibited 50% of DPPH radicals) was determined using linear regression analysis [44].

For the ABTS assay, the ABTS radical cation was generated by reacting ABTS with potassium persulfate, producing a green–blue solution with absorption peaks at 645, 734, and 815 nm. The EO was diluted in methanol and tested at various concentrations (0–2000 µg/mL). Then, 20 µL of EO was reacted with 980 µL of ABTS for 7 min in the dark at room temperature. Finally, absorbance was measured at 734 nm using a UV-VIS Genesys 150 spectrophotometer. The antioxidant activity was calculated as the percentage of radical inhibition. Trolox^®^ was used as a standard at a final concentration of 250 µg/mL (1 mM). IC_50_ values were determined from the inhibition curves via linear regression [45].

### 4.7. Acetylcholinesterase Inhibition of A. triphylla EO In Vitro

AChE activity was determined spectrophotometrically using Ellman’s method. The reaction mixture contained 40 µL of Tris buffer, 20 µL of EO, 20 µL of acetylthiocholine, and 100 µL of DTNB reagent. Donepezil hydrochloride with a calculated IC_50_ value of 12.40 ± 1.35 nM was used as a positive control. The samples were preincubated at 25 °C for 3 min with continuous shaking. Subsequently, 20 µL of AChE (0.5 U/mL) was added to initiate the reaction. The reaction was monitored using an EPOCH 2 microplate reader (BIOTEK, Winooski, VT, USA) at 405 nm for 60 min at 25 °C. Finally, 10 mg of the *A. triphylla* EO was dissolved in 1 mL of MeOH. Additional dilutions were added to obtain final concentrations of 1000, 100 and 10 µg/mL [46].

### 4.8. Brine Shrimp Cytoxicity Test

The lethality of the *A. triphylla* EOs was assessed using a lethality bioassay of salt shrimp larvae (*Artemia salina*). Standard and sample solutions of each EO (0.5–10 mg/mL) were prepared in 1% DMSO. In test tubes, 100 µL of each solution was added to 900 µL of synthetic seawater. Then, 20 larvae of *Artemia salina* were added. The test was repeated thrice for each concentration. After 24 h of incubation, the samples were observed using an Olympic SZX9 (Olympus, Tokyo, Japan) stereomicroscope. LC_50_ is defined as the lethal concentration that corresponds to 50% dead larvae and was determined for EO; potassium dichromate was used as a positive control.

### 4.9. Statistical Analysis

All experimental assays were performed in triplicate and the results are presented as mean ± standard deviation (SD). Antibacterial activity data (MIC values) were analyzed using two-way analysis of variance (ANOVA) followed by Sidak’s multiple comparison test to evaluate the differences among treatment groups and bacterial strains. Antioxidant activity (IC_50_ values for DPPH and ABTS) was compared with that of the Trolox standard using unpaired Student’s *t*-tests. For the cytotoxicity assay (*Artemia salina*), LC_50_ values were calculated using Probit regression analysis in R Studio (version 4.2.1), employing the MASS and drc packages. A *p*-value less than 0.05 was considered statistically significant in all tests. GraphPad Prism version 9.0 was used for all other statistical analyses.

## 5. Conclusions

The essential oil of *Aloysia triphylla* from Peru exhibited a unique chemical and enantiomeric profile that was rich in sesquiterpenes and monoterpenes, such as (*E*)-caryophyllene, *β*-pinene, and germacrene D. The EO demonstrated significant antibacterial activity against Gram-positive strains, weak antioxidant activity against DPPH and ABTS radicals, and moderate inhibitory activity against acetylcholinesterase, suggesting its potential neuroprotective effects. Its moderate cytotoxicity, as indicated by the *Artemia salina* assay, highlights the need for further toxicological assessment before therapeutic application. Overall, this study provides new insights into the bioactive potential of *A. triphylla* EO and supports its possible use in pharmaceutical, cosmetic, and functional product development.

## Figures and Tables

**Table 1:** Volatile components of *A. triphylla* EO identified on DB5-ms column.

No.	Compound	Retention Time	LRI ^a^	LRI ^b^	%Δ	% ± SD
1	*α*-Thujene	7.67	927	924	0.32	0.90 ± 0.15
2	*α*-Pinene	7.97	934	932	0.21	6.71 ± 0.85
3	*α*-Fenchene	8.67	951	945	0.63	0.36 ± 0.12
4	Sabinene	9.65	975	969	0.62	2.43 ± 0.38
5	*β*-Pinene	9.87	981	974	0.72	9.96 ± 0.95
6	myrcene	10.38	993	988	0.51	1.37 ± 0.35
7	δ-2-Carene	11.16	1011	1001	1.00	0.10 ± 0.04
8	Limonene	12.09	1031	1024	0.68	1.26 ± 0.42
9	Sylvestrene	12.20	1033	1025	0.78	2.97 ± 0.43
10	*β*-Phellandrene	12.30	1035	1025	0.98	0.30 ± 0.14
11	1,8-Cineole	12.41	1038	1026	1.17	2.92 ± 0.51
12	Not Identified	12.56	1041	--		0.17 ± 0.06
13	(*E*)-β-Ocimene	13.03	1051	1044	0.67	1.01 ± 0.40
14	γ-Terpinene	13.58	1063	1054	0.85	0.99 ± 0.16
15	Mentha-2,4(8)-diene	14.85	1090	1085	0.46	0.16 ± 0.03
16	Linalol	15.81	1111	1095	1.46	6.30 ± 0.90
17	trans-Sabinene hydrate	15.91	1113	1098	1.37	0.60 ± 0.13
18	1-Octen-3-yl acetate	16.03	1115	1110	0.45	1.27 ± 0.37
19	3-Octanol acetate	16.55	1126	1120	0.54	0.14 ± 0.05
20	iso-Menthone	18.52	1168	1158	0.86	0.20 ± 0.09
21	Rosefuran epoxide	19.39	1186	1173	1.11	0.23 ± 0.11
22	Terpinen-4-ol	19.66	1192	1174	1.53	0.67 ± 0.17
23	Pulegone	21.72	1237	1233	0.32	0.06 ± 0.02
24	Linalool acetate	22.46	1253	1254	0.08	1.27 ± 0.44
25	trans-Sabinene hydrate acetate	22.64	1257	1253	0.32	0.35 ± 0.10
26	trans-Pinocarvyl acetate	24.88	1305	1298	0.54	0.53 ± 0.16
27	trans-Carvyl acetate	26.60	1345	1339	0.45	0.04 ± 0.03
28	Silphinene	26.80	1349	1345	0.30	0.33 ± 0.13
29	*α*-Terpinyl acetate	27.15	1357	1346	0.82	0.08 ± 0.04
30	*α*-Ylangene	27.80	1372	1373	0.07	0.17 ± 0.07
31	*Z*-*β*-Damascone	28.45	1387	1386	0.07	0.92 ± 0.18
32	*β*-Cubebene	28.66	1391	1387	0.29	0.47 ± 0.11
33	*β*-Elemene	28.75	1393	1389	0.29	0.29 ± 0.09
34	Longifolene	29.45	1410	1407	0.21	0.37 ± 0.15
35	(*E*)-Caryophyllene	30.08	1425	1417	0.56	16.80 ± 1.00
36	*β*-Copaene	30.49	1435	1430	0.35	0.79 ± 0.14
37	*α*-Guaiene	30.84	1443	1437	0.42	0.21 ± 0.08
38	*α*-Humulene	31.59	1461	1452		2.54 ± 0.29
39	allo-Aromadendrene	31.77	1466	1458	0.55	0.17 ± 0.05
40	cis-Muurola-4(14),5-diene	31.88	1468	1465	0.20	0.24 ± 0.12
41	γ-Muurolene	32.43	1481	1478	0.20	1.16 ± 0.39
42	Germacrene D	32.70	1488	1480	0.54	10.00 ± 0.95
43	γ-Amorphene	33.17	1499	1495	0.27	0.53 ± 0.18
44	Bicyclogermacrene	33.30	1502	1500	0.13	2.75 ± 0.45
45	trans-*β*-Guaiene	33.43	1506	1502	0.27	0.66 ± 0.19
46	(*E*, *E*)-*α*-Farnesene	33.62	1510	1505	0.33	0.35 ± 0.11
47	γ-Cadinene	34.03	1521	1513	0.53	0.73 ± 0.13
48	δ-Cadinene	34.21	1525	1522	0.20	1.71 ± 0.22
49	trans-Calamenene	34.39	1530	1521	0.59	0.22 ± 0.10
50	*α*-Cadinene	34.99	1545	1537	0.52	0.11 ± 0.04
51	(*E*)-Nerolidol	36.02	1571	1561	0.64	0.10 ± 0.03
52	Spathulenol	36.82	1591	1577	0.89	1.52 ± 0.41
53	Caryophyllene oxide	36.95	1595	1582	0.82	3.33 ± 0.43
54	*β*-Copaen-4-*α*-ol	37.07	1598	1590	0.50	0.18 ± 0.06
55	Not Identified	37.13	1599	--		0.16 ± 0.07
56	Salvial-4(14)-en-1-one	37.41	1607	1594	0.82	0.25 ± 0.08
57	Humulene epoxide II	38.08	1625	1608	1.06	0.33 ± 0.16
58	Muurola-4,10(14)-dien-1-β-ol	38.42	1634	1630	0.25	0.07 ± 0.03
59	allo-Aromadendrene epoxide	39.01	1649	1639	0.61	0.13 ± 0.05
60	Cedr-8(15)-en-9-*α*-ol	39.23	1655	1650	0.30	0.11 ± 0.06
61	Selin-11-en-4-*α*-ol	39.33	1658	1658	0.00	0.08 ± 0.02
62	7-epi-*α*-Eudesmol	39.88	1672	1662	0.60	0.35 ± 0.12
63	Eudesma-4(15),7-dien-1β-ol	40.68	1694	1687	0.41	0.06 ± 0.04
64	Guaiol acetate	41.35	1712	1725	0.75	0.18 ± 0.08
Monoterpene hydrocarbons (%)						25.10 ± 1.00
Oxygenated monoterpenes (%)						31.12 ± 0.90
Sesquiterpene hydrocarbons (%)						37.40 ± 0.45
Oxygenated sesquiterpenes (%)						6.66 ± 0.41
Other compounds/unidentified						2.65 ± 0.08
Total identified						99.84%

LRI ^a^ = calculated linear retention index; LRI ^b^ = linear retention index from reference; mean ± SD (*n* = 3); %Δ = percentage difference.

**Table 2:** Enantiomeric distribution of *A. triphylla* EO on a cyclodextrin column. Mean ± SD (*n* = 3). *e.e.*= enantiomeric excess.

Enantiomer	LRI	Enantiomeric Distribution (%)	*e.e.* (%)
(1*S*,5*S*)-(−)-*α*-pinene	928	95.76	91.51
(1*R*,5*R*)-(+)-*α*-pinene	930	4.25	
(1*S*,5*S*)-(−)-*β*-pinene	979	55.48	11.05
(1*R*,5*R*)-(+)-*β*-pinene	980	44.43	
(1*S*,5*S*)-(–)-sabinene	991	100	100
(*R*)-(+)-limonene	1069	100	100
(*R*)-(–)-linalool	1182	57.53	15.06
(*S*)-(+)-linalool	1192	42.46	
(*R*)-(+)-germacrene D	1461	100	100

**Table 3:** Determination of the minimum inhibitory concentrations (MIC) of *A. triphylla* EO and ciprofloxacin using the colorimetric microdilution method.

Microorganism	MIC		Two-Way ANOVA Test	Sidak’s Multiple Comparison Test
	Essential Oil	Positive Control		
	*A. triphylla* (µg/mL)	Ciprofloxacin (µg/mL)		
*S. aureus*	80.0 ± 2.00	15.0 ± 0.05	*p* < 0.0001	*p* < 0.0001
*B. subtilis*	5.0 ± 1.00	0.625 ± 0.01		*p* < 0.0001
*E. coli*	320.0 ± 0.58	0.625 ± 0.01		*p* < 0.0001
*P. aeruginosa*	>500	1.25 ± 0.05		*p* < 0.0001

**Table 4:** Antioxidant activity of *A. triphylla* EO. Mean ± SD (*n* = 3).

Antioxidant Assay		Essential Oil	Trolox	*p* Value (T-Student’s Test)
DPPH	µmol TE/g	0.459 ± 0.002	-	*p* < 0.0001
	IC_50_ (µg/mL)	7720.0 ± 12.23	3.81 ± 0.002	
ABTS	µmol TE/g	0.462± 0.001	-	*p* < 0.0001
	IC_50_ (µg/mL)	4648.6 ± 1.25	2.31 ± 0.001	

**Table 5:** Cytotoxicity of *A. triphylla* EO against *Artemia salina*. Mean ± SD (*n* = 3).

Substances	LC_50_ µg/mL ± SD
Essential oil	964.0 ± 20.6
Potassium dichromate	70.5 ± 2.2
Sea water with DMSO 1%	No toxicity

## Data Availability

The datasets generated during and/or analyzed during the current study are available from the corresponding author upon reasonable request.

## Supplementary Materials

The following supporting information can be downloaded at: https://www.mdpi.com/article/10.3390/molecules30132849/s1, Figure S1: Chromatogram profile of *Aloysia triphylla* Royle essential oil; Figure S2: Chiral separation of (1*S*,5*S*)-(-)-α-pinene and (1*R*,5*R*)-(+)-α-pinene standards. Figure S3: Chiral separation of (1*S*,5*S*)-(-)-β-pinene and (1*R*,5*R*)-(+)-β-pinene standards. Figure S4: Chiral separation of (1*R*,5*R*)-(+)-sabinene and (1*S*,5*S*)-(–)-sabinene standards. Figure S5: Chiral separation of (*S*)-(-)-limonene and (*R*)-(+)-limonene standards. Figure S6: Chiral separation of (*R*)-(–)-linalool and (*S*)-(+)-linalool standards. Figure S7: Chromatogram of (*R*)-(+)-germacrene D standard. Figure S8: Chromatogram of η-alkanes (C9-C23) injected in DB5-ms column.
