# Supplementary material for: Unveiling the Chemical Composition, Enantiomeric Profile, Antibacterial, Anticholinesterase and Antioxidant Activity of the Essential Oil of Aloysia triphylla Royle
Source: Molecules. 2025 Jul 3;30(13):2849. doi: 10.3390/molecules30132849 (PMC12251356; doi:10.3390/molecules30132849)

Figure S1: Chromatogram profile of *Aloysia triphylla* Royle essential oil; **1** (1*S*,5*S*)-(-)-α-pinene (RIcal: 928); **2** (1*R*,5*R*)-(+)-α-pinene (RIcal: 930); **3** (1*S*,5*S*)-(-)-β-pinene (RIcal: 978); **4** (1*R*,5*R*)-(+)-β-pinene (RIcal: 980); **5** (1*S*,5*S*)-(–)-sabinene (RIcal: 991); **6** (*R*)-(+)-limonene (RIcal: 1069); **7** (*R*)-(-)-linalool (RIcal: 1182); **8** (*S*)-(+)-linalool (RIcal: 1192); **9** (*R*)-(+)-germacrene D (RIcal: 1461)

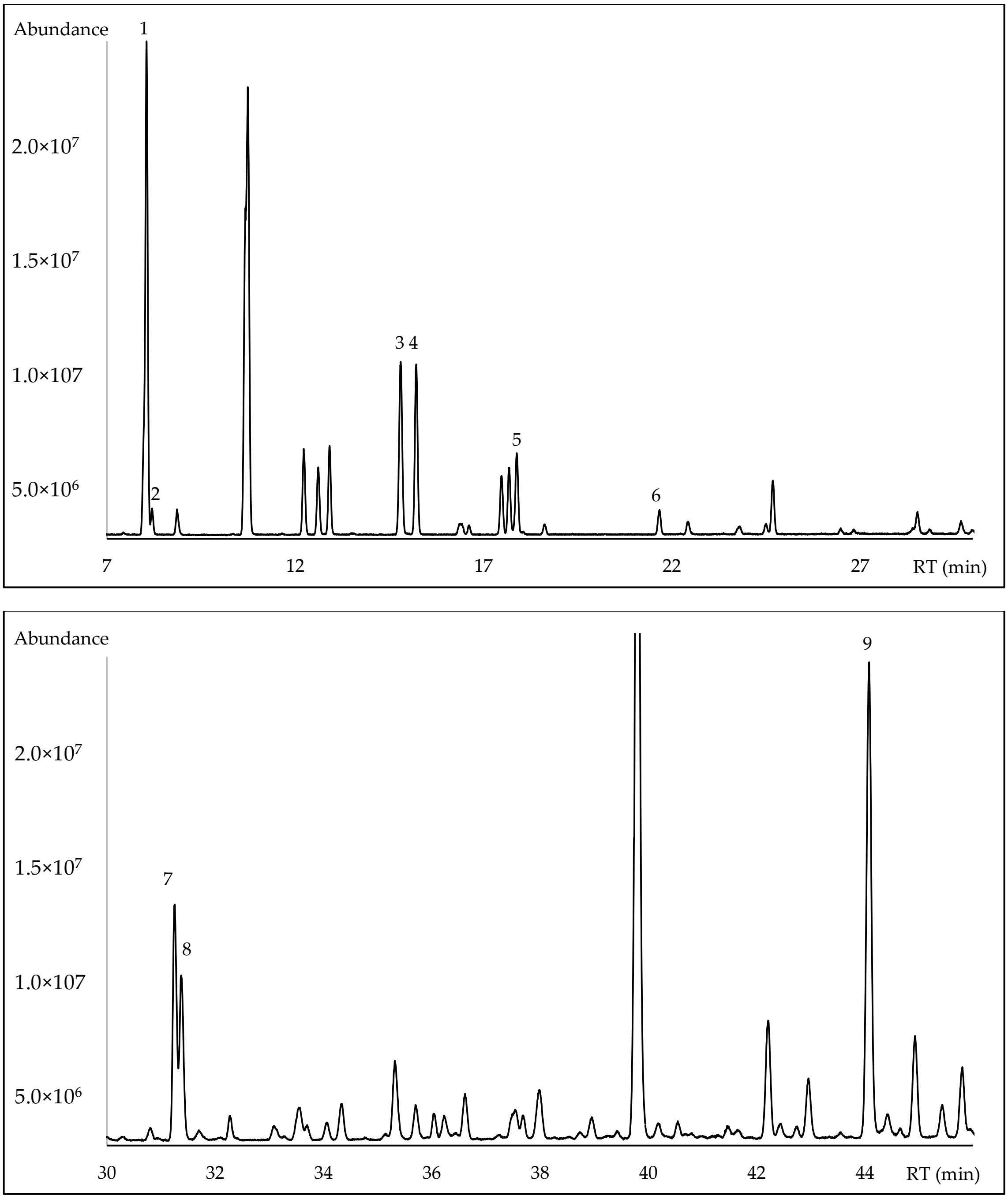

Figure S2: Chiral separation of (1*S*,5*S*)-(-)-*α*-pinene and (1*R*,5*R*)-(+)-*α*-pinene standards.

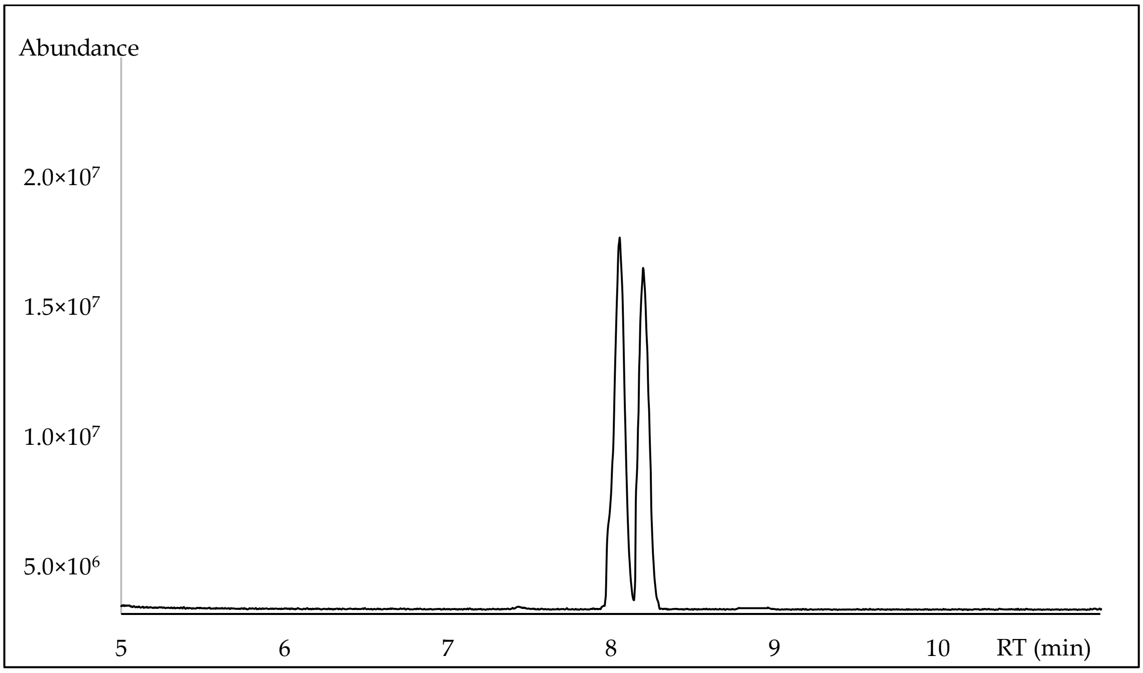

Figure S3: Chiral separation of (1*S*,5*S*)-(-)-*β*-pinene and (1*R*,5*R*)-(+)-*β*-pinene standards.

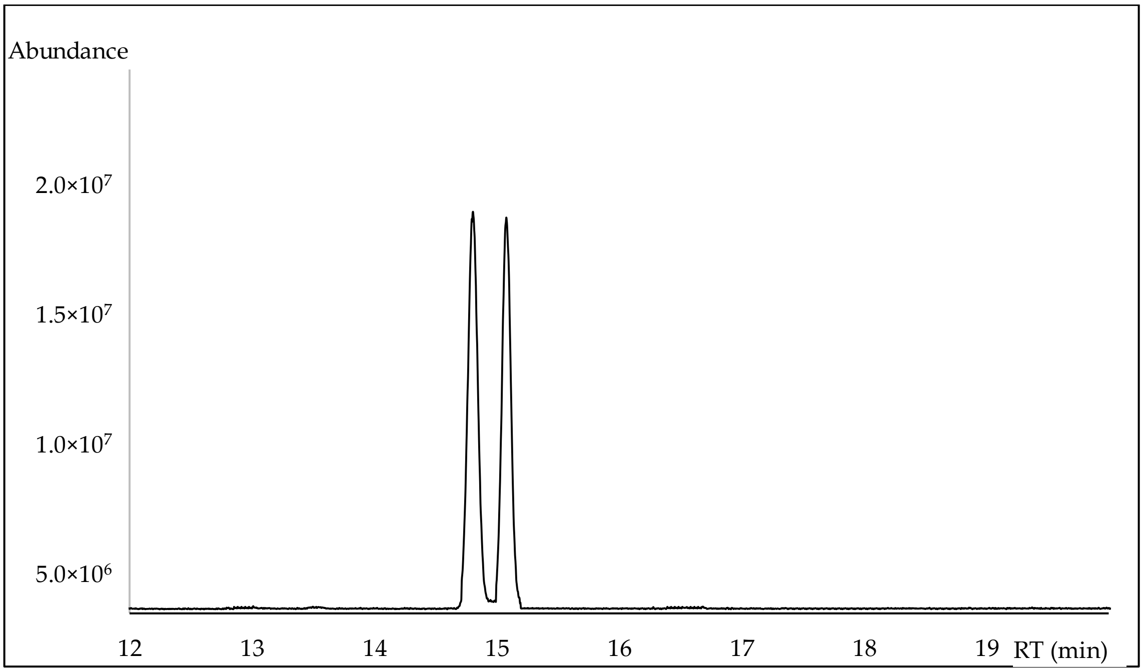

Figure S4: Chiral separation of (1*R*,5*R*)-(+)-sabinene and (1*S*,5*S*)-(–)-sabinene standards.

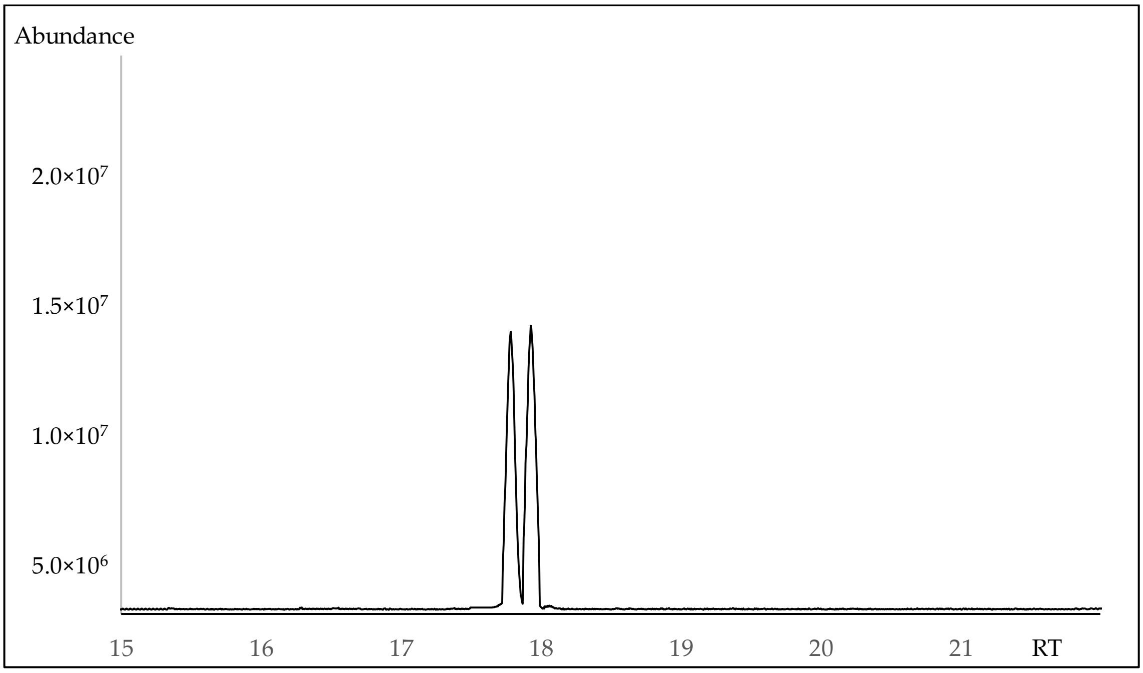

Figure S5: Chiral separation of (*S*)-(-)-limonene and (*R*)-(+)-limonene standards.

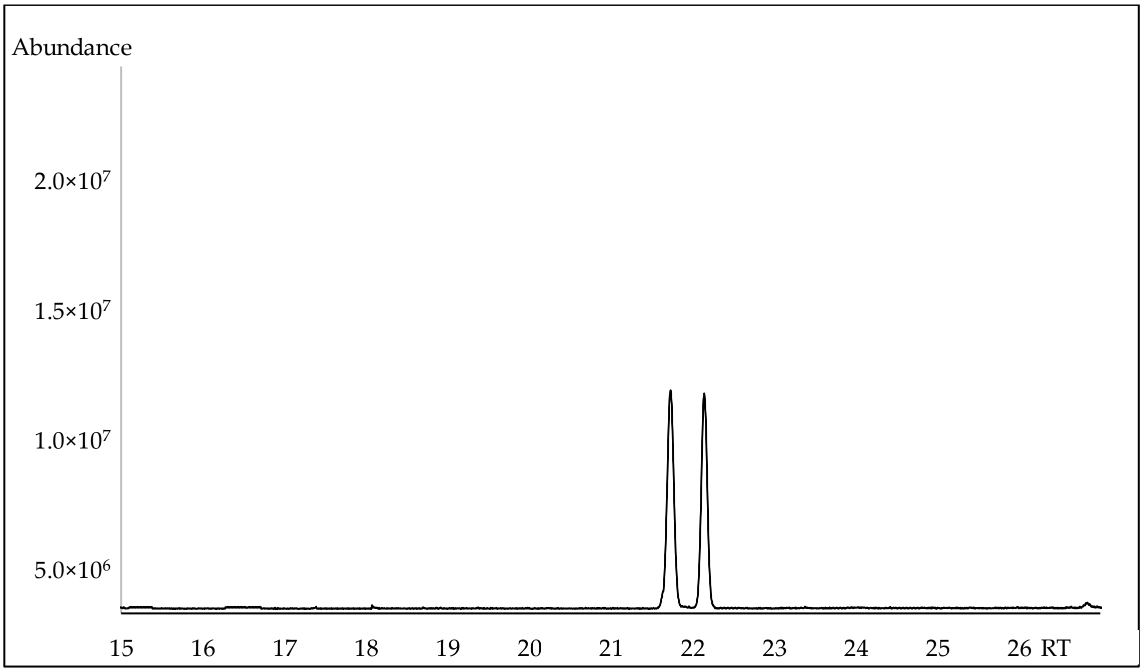

Figure S6: Chiral separation of (*R*)-(–)-linalool and (*S*)-(+)-linalool standards.

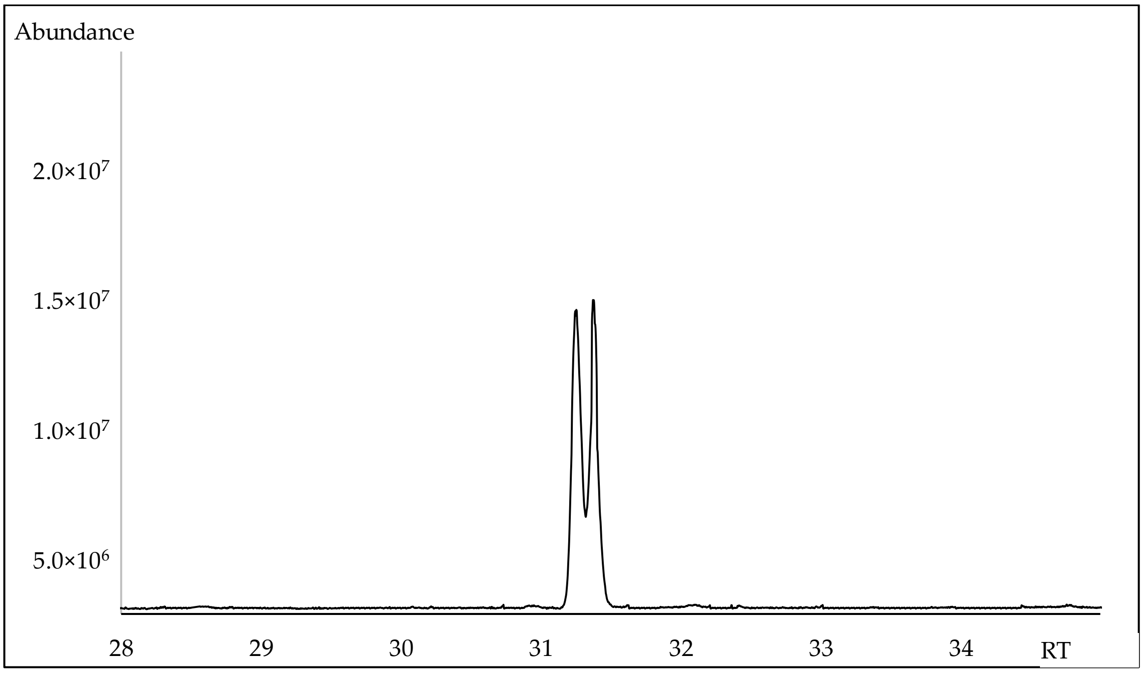

Figure S7: Chromatogram of (*R*)-(+)-germacrene D standard.

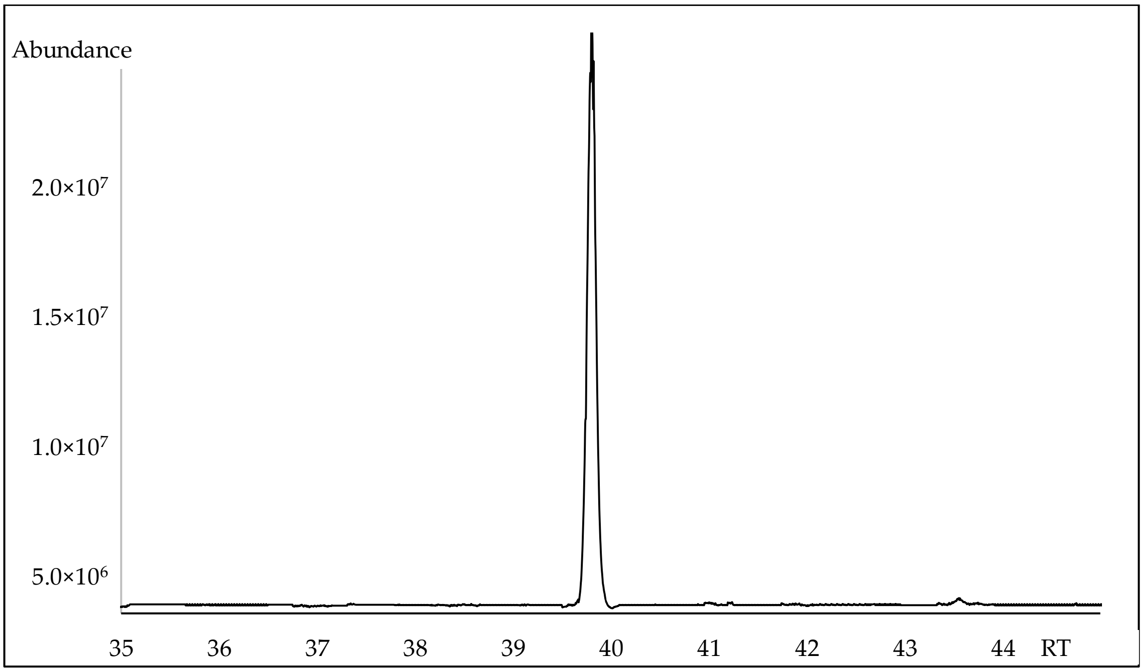

Figure S8: Chromatogram of η-alkanes (C_9-C_{23}) injected in DB5-ms column

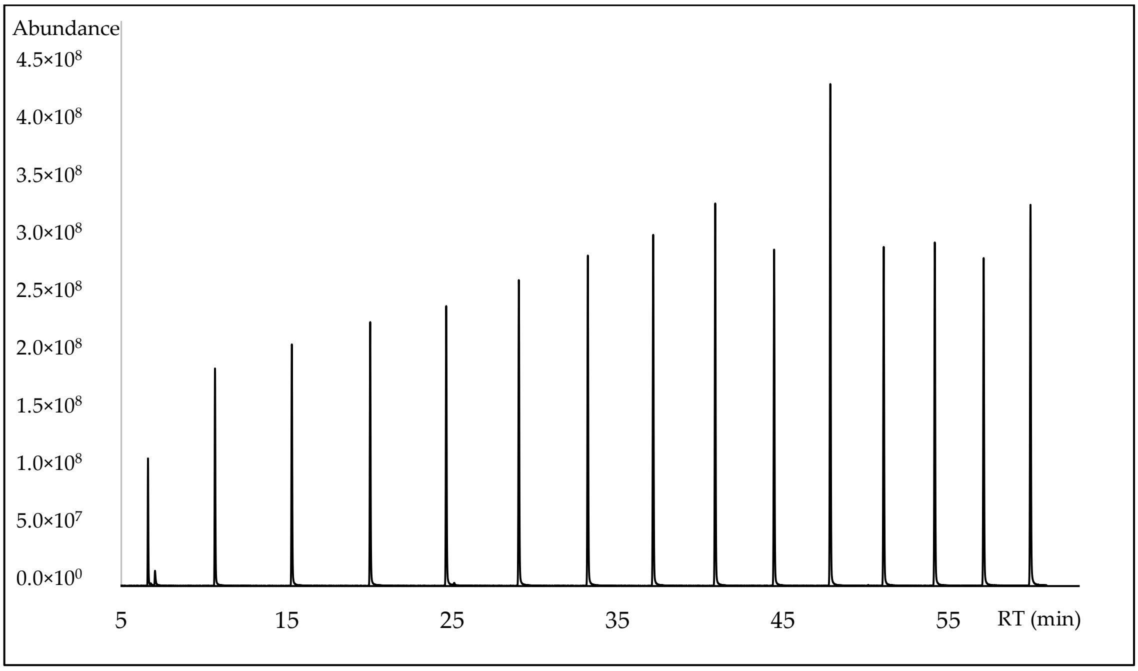

Supplement: Supplementary file 1 [file molecules-30-02849-s001.zip › suplementary material Aloysia triphylla (1).pdf]
